# Draft Genome Sequence of Non-O1 and Non-O139 *Vibrio cholerae* Strain VCC19

**DOI:** 10.1128/genomeA.01094-14

**Published:** 2014-11-06

**Authors:** Pablo Caracciolo Gomes de Sá, Miriam Lopes Da Silva, Adriana Ribeiro Carneiro, Jaqueline Conceição Meireles Gomes, Larissa Maranhão Dias, Jorianne Thyeska Castro Alves, Adonney Allan De Oliveira Veras, Rafael Azevedo Baraúna, Diego Assis Das Graças, Maria Helena Matté, Maria Ines Zanolli Sato, Elayse Maria Hachich, Glavur Rogério Matté, Rommel Thiago Jucá Ramos, Artur Silva

**Affiliations:** a Instituto de Ciências Biológicas, Universidade Federal do Pará, Belém, Pará, Brazil; b Laboratório de Saúde Pública, Departamento de Prática de Saúde Pública, Faculdade de Saúde Pública, Universidade de São Paulo, São Paulo, Brazil; c CETESB−Companhia Ambiental do Estado de São Paulo, São Paulo, Brazil

## Abstract

*Vibrio cholerae* O1 is the causative agent of cholera and is ubiquitous in the aquatic environment, while *V. cholerae* strains non-O1 and non-O139 are recognized as causative agents of sporadic and localized outbreaks of diarrhea. Here, we report the complete sequence of a non-O1 and non-O139 *V. cholerae* strain (VCC19), which was isolated from the environment in Brazil. The sequence includes the integrative conjugative element (ICE). This paper is the first report of the presence of such an element in a *V. cholerae* strain isolated in Brazil.

## GENOME ANNOUNCEMENT

*Vibrio cholerae* is a Gram-negative, curved, rod-shaped bacteria, and it is ubiquitous in the aquatic environment. *V. cholerae* O1 is the causative agent of cholera, which is a diarrheic disease that is produced due to the presence of a virulence gene cassette, which is recognized as the main factor that contributes to the pathogenicity in the toxigenic strains of O1 and O139 serotypes (1). Non-O1 and non-O139 *V. cholerae* strains are recognized as causative agents of sporadic and localized outbreaks of diarrhea. Studies have demonstrated that non-O1 and non-O139 *V. cholerae* strains may be involved in the emergence of *V. cholerae* O139 by the acquisition of virulence genes from *V. cholerae* O1 (1). Integrative conjugative element (ICEs) originally isolated in *V. cholerae* contribute to the response of the bacteria to environmental adversities, spreading resistance genes in different genera of bacteria in several countries (2, 3). A comparison of the constitutive genes of one SXT element with others obtained worldwide helps to determine the origin of the strain, elucidating the epidemiological pathway that is related to the reemergence of cholera in one country, where the disease was absent for many years (2). In this paper, we present the genome sequence of a non-O1 and non-O139 *V. cholerae* strain (VCC19) that possesses the SXT (ICE) element; this strain was isolated from an environmental sample obtained in São Paulo, Brazil, in 2001 and were subjected to whole-genome shotgun sequencing. This genome might explain the epidemiological pathway of the reemergence of the cholera epidemic in Brazil.

Chromosomal DNA was extracted from a non-O1 and non-O139 *V. cholerae* strain (VCC19) by the cetyltrimethylammonium bromide method, as described by Ausubel et al. (4). The full genome of this bacterium was sequenced with the Ion Torrent PGM sequencer using a mate-pair library with a 3-kb insert size, which helps to represent repetitive regions in the genome assembly (5). The sequences were extracted from an SFF file with the script “sff_extract_0_2_13” (http://bioinf.comav.upv.es/sff_extract); this action resulted in a total of 6,985,480 reads (1,781,040,841 bp), which represent a total coverage of ~441× when considering both chromosomes I and II (GenBank accession numbers AE003852.1 and AE003853.1).

The reads were assembled by a *de novo* approach using the software Mira (6), which produced 54 contigs. The sequences were annotated by RAST (7). The draft genome has 4,134,889 bp, 3,936 coding sequences (CDSs), 63 tRNAs, and 24 rRNAs.

### Nucleotide sequence accession number.

The non-O1 and non-O139 *V. cholerae* strain (VCC19) draft genome sequence has been deposited in GenBank under the accession number ATEV00000000.
